# Is There a Relationship Between Bovine Tuberculosis (bTB) Herd Breakdown Risk and *Mycobacterium avium* subsp. *paratuberculosis* Status? An Investigation in bTB Chronically and Non-chronically Infected Herds

**DOI:** 10.3389/fvets.2019.00030

**Published:** 2019-02-14

**Authors:** Andrew W. Byrne, Jordon Graham, Georgina Milne, Maria Guelbenzu-Gonzalo, Sam Strain

**Affiliations:** ^1^Veterinary Science Division, Agri-food and Biosciences Institute, Belfast, United Kingdom; ^2^School of Biological Sciences, Queen's University Belfast, Belfast, United Kingdom; ^3^Animal Health Ireland, Carrick on Shannon, Ireland; ^4^Animal Health and Welfare Northern Ireland, Dungannon, United Kingdom

**Keywords:** bovine TB, Johne's disease, veterinary epidemiology, co-infection, infectious disease control, mycobacteria

## Abstract

**Background:** Bovine tuberculosis (bTB; *Mycobacterium bovis*) remains a significant problem in a number of countries, and is often found where *M. avium* subsp. *paratuberculosis* (MAP) is also present. In the United Kingdom, bTB has been difficult to eradicate despite long-term efforts. Co-infection has been proposed as one partial mechanism thwarting eradication.

**Methods:** A retrospective case-control study of 4,500 cattle herds in Northern Ireland, where serological testing of cattle for MAP, was undertaken (2004–2015). Blood samples were ELISA tested for MAP; infection of *M. bovis* was identified in herds by the comparative tuberculin test (CTT) and through post-mortem evidence of infection. Case-herds were those experiencing a confirmed bTB breakdown; control-herds were not experiencing a breakdown episode at the time of MAP testing. A second model included additional testing data of feces samples (culture and PCR results) to better inform herd MAP status. Multi-level hierarchical models were developed, controlling for selected confounders. A sensitivity analysis of the effect of MAP sample numbers per event and the prior timing of tuberculin-testing was undertaken.

**Results:** 45.2% (*n* = 250) of case observations and 36.0% (3,480) of control observations were positive to MAP by ELISA (45.8% and 36.4% when including ancillary fecal testing, respectively). Controlling for known confounders, the adjusted odds ratio (aOR) for this association was 1.339 (95%CI:1.085–1.652; including ancillary data aOR:1.356;95%CI:1.099–1.673). The size-effect of the association increased with the increasing number of samples per event used to assign herd MAP status (aOR:1.883 at >2 samples, to aOR:3.863 at >10 samples), however the estimated CI increased as *N* decreased. 41.7% of observations from chronic herds were MAP serology-positive and 32.2% from bTB free herds were MAP positive (aOR: 1.170; 95%ci: 0.481–2.849).

**Discussion:** Cattle herds experiencing a bTB breakdown were associated with increased risk of having a positive MAP status. Chronic herds tended to exhibit higher risk of a positive MAP status than bTB free herds, however there was less support for this association when controlling for repeated measures and confounding. MAP co-infection may be playing a role in the success of bTB eradiation schemes, however further studies are required to understand the mechanisms and to definitively establish causation.

## Introduction

Chronic mycobacterial infections are some of the most challenging pathogens to eradicate at national and international scales, requiring large-scale long-term coordinated eradication efforts (1–3). For the United Kingdom, bovine tuberculosis (caused by infection with the pathogen *Mycobacterium bovis*) remains one of the highest priority pathogens affecting cattle farming industries, and one of the most costly pathogens to control to the state (4, 5). Indeed, in Northern Ireland alone, the annual cost of the eradication program in recent years has exceeded £30 million (http://www.tbhub.co.uk/tb-policy/northern-ireland/). Eradication in the UK is primarily predicated on the accurate identification of the pathogen within herds using a screening comparative tuberculin test (using bovine and avian tuberculins) to allow the slaughter of test positive animals. Subsequent repeated tuberculin testing is undertaken, and where there is evidence of within herd transmission, ancillary testing using the more sensitive interferon gamma test, and removal of additional test positive animals may be undertaken (6). The sensitivity of the screening test (single intradermal cervical comparative tuberculin (SICCT) test) is known to be moderate and can be variable in performance (7–10). A further complication is the presence of a known wildlife reservoir species that can harbor widespread and high levels of infection (11, 12), and which cull trials have provided evidence to suggest are involved in the risk of infection within cattle herds (13, 14). Despite these issues, modeling studies suggest that the mean reproductive number of the system (R0), which determines whether infection will be maintained in populations in perpetuity, is only slightly above 1 (15–17). Therefore, only small positive changes to the eradication program could bring the trajectory below 1, which would in the long-term lead to eradication (5).

*M. avium* subsp. *paratuberculosis* (MAP) is the causative agent of Johne's disease, another significant mycobacterial pathogen of cattle, which can impact production and thus lead to economic loses to farmers (18). Unlike bTB, Johne's disease is not a statutory pathogen in Britain and Ireland, however, it is considered a priority non-regulatory pathogen of concern to farmers (19). There is evidence that co-infection of MAP can impact on the immunological response to tuberculins during statutory bTB skin testing and interferon gamma tests (20–24). This is due to cross-reactivity, primarily on the avian tuberculin (which is derived from *M. avium*), but also impacts the reaction size on the bovine injection site (21, 25). Such cross-reactivity effects can lead to misdiagnosis (7, 26), allowing for TB positive animals to be mistakenly retained within herds. One could hypothesize that these processes may have impacts on the ability to clear infection once detected within herds (21), and subsequently impacts the duration of breakdown episodes. Unsurprisingly, the exposure to *M. bovis* can also have an impact on the performance of diagnostics for MAP (27). Therefore, it can be challenging to assign mechanistic inference, as the timing of infections and the testing regimes will have an impact on patterns that emerge from co-infected herds. For example, evidence suggests that tuberculin testing may impact on serum and milk ELISA testing for MAP, causing an increased risk of false positive disclosure (25, 28). Despite this, there is some epidemiological evidence that co-infection can hamper the ability to accurately identify infected animals and to clear infection from herds when the primary aim is either to eradicate bTB [e.g., (23)] or MAP [e.g., (27)].

In this study, we undertook a retrospective case-control study to investigate the hypothesis that there was an association between MAP herd status and the probability of a herd experiencing a confirmed bTB breakdown episode. We also wanted to investigate whether there were any patterns of co-infection and the longer-term bTB status of herds. We hypothesized that herds with “chronic” (i.e., long-term and recurrent problem herds) TB infection may be more likely to have evidence of co-infection than herds that did not experience chronic infections.

## Methods

A retrospective, case-control study of cattle herds in Northern Ireland (United Kingdom) was undertaken (2004–2015). The primary dataset utilized during the study included serological testing data generated from surveillance activities, but primarily from part of a cattle health scheme in Northern Ireland undertaken by the Agri-food and Biosciences Institute (AFBI; www.afbi.gov.uk). These data included all blood (serological) tests undertaken during voluntary submissions, as well as those made by members of AFBI Cattle Health Scheme (CHS). Therefore, inclusion criteria restricted herds to those sampled and which voluntarily participated in MAP control schemes in Northern Ireland. It should be noted that all samples were handled and tested in the same way within AFBI irrespective of the scheme from which samples were received.

The AFBI CHS is a voluntary scheme which provides a structured approach to the control of five endemic diseases of cattle including Johne's disease. The scheme is licensed by Cattle Health Certification Standards (CHeCS) which provides the standards for licensed health schemes within the UK and Ireland. Herds enrolled in the Johne's programme have to carry out an annual herd screen consisting on blood testing all animals 2 years of age or older within the herd. In low seroprevalence herds, seropositive animals could be re-tested by fecal culture or PCR to determine the status (below for more). Introduced animals had to be blood/fecal sampled in isolation prior to joining the herd independently of the age.

Bloods were primarily sampled during concurrent statutory test sampling [i.e., TB testing or brucellosis monitoring; (29)]. All veterinarians and herd owners involved in CHS and other voluntary MAP testing with AFBI were given advice to avoid cross-reactions following intradermal tuberculin testing by either blood sampling on the first day of the test or waiting 3 months. These samples were date stamped within the dataset with the submission to the laboratory, with the statutory key performance indicator (KPI) ensuring that samples were processed within a week or less (S. Verner, pers. comm.). It should be noted that not all samples were taken at the same time as those for ancillary tests, with a risk that the time between sampling and sample submission within the field may have varied (e.g., the fecal samples, see below). However, we have no evidence to support the hypothesis that there would be any systematic bias in submission times. We assumed this random error contributed to the uncertainty in the error in our models, but potentially erring the models toward the null.

The testing was undertaken in the Diagnostic Surveillance and Investigation Branch (DSIB) at AFBI, Stormont. Blood samples were tested using the IDEXX Paratuberculosis Screening AB ELISA Test (formerly Pourquier) as per the manufacturer's recommendations. This test was accredited to ISO17025 standard. Additional information on kits used and laboratory procedures are presented in the Supplementary Material. There were also a small number of feces samples taken from animals within herds where MAP was suspected (after a positive ELISA result or clinical suspicion) or from animals added to AFBI CHS herds in the Johne's programme. These samples were tested for MAP via culturing of the pathogen in the laboratory (30), or using PCR methodologies (see Supplementary Material). These ancillary data were added to the serological dataset to improve the ability to designate a MAP herd status.

For the purposes of the present study, for each herd, a MAP status was attributed to the herd at the date of MAP testing. If any positive result for MAP was disclosed from the dataset, then the herd was attributed a “positive status” for that test event (note, this is not necessarily the case for the program, where a herd is not considered positive with a single seropositive result due to imperfect test specificity). Herds could be represented in the dataset on multiple occasions as repeated MAP tests occurred over the time series.

The Animal and Public Health and Information System (APHIS) maintained by the Department of Agriculture, Environment and Rural Affairs (DAERA, Northern Ireland) was used to gain information on herd bovine tuberculosis status, as well as deriving data on herd type, whether the herd held a milk license, which DVO the herd was assigned to, the number of animals moving into a herd per annum (buying in), and for estimating herd size.

## Study Design and Modeling

The outcome variable of interest was the binary bTB status of herds at the time of the MAP testing event. The bTB status of the herd was based on breakdown episodes (31, 32). An episode was a period of time a herd was undergoing a breakdown, and was defined as the period from the first disclosing test to the last test at which the herd was released from restriction [for more on this classification see (31, 32)]. Only episodes whereby TB was post-mortem laboratory confirmed during an episode were included in this episode file (these are either multiple reactor breakdowns and/or breakdowns triggered by the presence of a TB lesion). Laboratory confirmation in Northern Ireland includes histopathology, culture, and/or strain/genotyping [spoligotyping and Variable Number Tandem Repeat genotyping; (33)]. This episode file then allowed for each herd to be classified as either “within episode,” which was our cases, and “not experiencing a breakdown” which was our control observations at the time of the MAP test event.

The primary explanatory variable was herd MAP status at the test event level based on the serological ELISA test classification. A secondary model also included ancillary data (feces) to classify herds with regards MAP. It is important to note that MAP and bTB are currently not co-managed in Northern Ireland as part of the bTB eradication scheme.

Bovine TB has a complex epidemiology, and we wanted to control for the most significant confounders of bTB risk in Northern Ireland but limiting the number of parameters in our model to ensure the model was reasonably parsimonious. Given previous experience in Northern Ireland [e.g., (6)] and published reviews of the literature [e.g., (34)], we made the *a-priori* decision to control for herd type [measured in two ways (Table 1)], herd size, number of animals bought in to the herd during the test year, and region (based on 10 District Veterinary Office administrative areas), while controlling for the non-independence of repeated observations within the same herd as a baseline model. We also tested whether there was any evidence of significant 2-way interactions between MAP status and (i) herd type (ii) herd size (iii) buying in, and also whether there was an interaction between herd type and (i) herd size (ii) buying in. Year was additionally tested for inclusion as a linear predictor, as a quadratic function (year^2^), as the natural log [log(year)], and a triennial categorical variable (dummy variable with four categories). Details of the variable are presented in Table 1. It should be noted that two herd-type variables were considered, HERD_TYPE and MILK_LICENSE. HERD_TYPE contained information about the enterprise type classification used in APHIS but also had some missing classifications, whereas MILK_LICENSE only allowed for binary classification of herds to those with/without a milk license irrespective of main enterprise type recorded within the APHIS dataset.

**Table 1:** Cross-tabulations and univariable analysis of the relationship between explanatory variables and bovine TB episode cases (positive) and controls (negative) in cattle herds in Northern Ireland.

	**TB negative**	**TB positive**	**Total**	**OR**	**Lower 95%CI**	**Upper 95%CI**
**MAP SERUM ELISA**						
Negative	6,186	303	6,489	ref.		
%	*95.33*	*4.67*	*100*			
Positive	3,480	250	3,730	1.467	1.234	1.743
%	*93.3*	*6.70*	*100*			
**MAP STATUS (SERUM+FECES)**						
Negative	6,152	300	6,452	ref.		
%	*95.35*	*4.65*	*100*			
Positive	3,514	253	3,767	1.476	1.243	1.754
%	93.28	6.72	100			
**YEAR**						
*2004–2006*	515	31	546	ref.		
%	*94.32*	*5.68*	*100*			
*2007–2009*	749	46	795	1.020	0.618	1.683
%	*94.21*	*5.79*	*100*			
*2010–2012*	3,716	225	3,941	1.006	0.664	1.523
%	*94.29*	*5.71*	*100*			
*2013–2015*	4,686	251	4,937	0.890	0.589	1.344
%	*94.92*	*5.08*	*100*			
**HERD_TYPE**						
Beef	4,363	188	4,551	ref.		
%	*95.87*	*4.13*	*100*			
Dairy	3,557	300	3,857	1.957	1.623	2.361
%	*92.22*	*7.78*	*100*			
Other/missing	1,746	65	1,811	0.864	0.648	1.152
%	*96.41*	*3.59*	*100*			
**MILK LICENSE**						
Yes	6,446	280	6,726	1.952	1.643	2.318
%	*95.84*	*4.16*	*100*			
No	3,220	273	3,493	ref.		
%	*92.18*	*7.82*	*100*			
**dvo_code**						
1	762	47	809	ref.		
*Armagh*	*94.19*	*5.81*	*100*			
2	360	11	371	0.495	0.254	0.966
*Ballymena*	*97.04*	*2.96*	*100*			
3	1,049	58	1,107	0.896	0.603	1.332
*Coleraine*	*94.76*	*5.24*	*100*			
4	1,355	61	1,416	0.730	0.494	1.079
*Dungannon*	*95.69*	*4.31*	*100*			
5	1,257	64	1,321	0.825	0.561	1.216
*Enniskillen*	*95.16*	*4.84*	*100*			
6	264	9	273	0.553	0.267	1.143
*Larne*	*96.7*	*3.3*	*100*			
7	366	21	387	0.930	0.548	1.579
*Derry/Londonderry*	*94.57*	*5.43*	*100*			
8	1,293	89	1,382	1.116	0.775	1.607
*Newry*	*93.56*	*6.44*	*100*			
9	1,076	90	1,166	1.356	0.942	1.953
*Newtownards*	*92.28*	*7.72*	*100*			
10	1,884	103	1,987	0.886	0.621	1.264
*Omagh*	*94.82*	*5.18*	*100*			
**BUYING IN (MEAN; RANGE)**						
Q1 (0.389; < 2)	2,711	152	2,863	ref.		
%	*94.69*	*5.31*	*100*			
Q2 (3.205; 2–5)	2,162	96	2,258	0.792	0.610	1.029
%	*95.75*	*4.25*	*100*			
Q3 (10.416; 6–17)	2,481	110	2,591	0.791	0.615	1.017
%	*95.75*	*4.25*	*100*			
Q1 (75.280; >17)	2,312	195	2,507	1.504	1.208	1.873
%	*92.22*	*7.78*	*100*			
**HERD SIZE (MEAN; RANGE)**						
Q1 (23.451; < 42)	2,570	36	2,606	ref.		
%	*98.62*	*1.38*	*100*			
Q2 (63.924; 42–90)	2,435	85	2,520	2.492	1.681	3.694
%	*96.63*	*3.37*	*100*			
Q2 (133.823; 91–194)	2,386	161	2,547	4.817	3.342	6.943
%	*93.68*	*6.32*	*100*			
Q1 (370.628; >195)	2,275	271	2,546	8.504	5.979	12.095
**%**	*89.36*	*10.64*	*100*			

Simple unadjusted associations between variables was assessed using logistic regression. For the multivariable models, the data were fitted to a binary logit random effect regression model (random effect = herd_id). All independent variables were included in all models, with the exception of HERD_TYPE and MILK_LICENSE, which were entered into competing models and the model with the lowest Akaike's Information Criteria (AIC) value was considered the better fitting model. Where the difference in AIC was < 2, models were considered equivalent (35). The random effect model was compared with a logistic regression to assess if there was significant clustering within the dataset, with a likelihood-ratio test of the intraclass correlation (ICC; ρ) being statistically different from zero. The classification ability of the model was assessed by calculating the Area under the ROC (AUC), with a model with >0.7 considered exhibiting reasonable performance.

## Sensitivity Analysis

The mean number of animals sampled for Johne's Disease per herd per event was 10.02 (SD: 34.091), however there was a large variance in the distribution with the median sample of 1. The sensitivity of ELISA tests for MAP can be low [e.g., 26%; (36)], and can depend on the infectious stage of the host (37), and thus can also be highly variable in terms of performance. Recent work from the UK has suggested that moving toward a more probabilistic interpretation of MAP ELISA tests based on multiple (repeat) samples could improve the ability to assign infection status (38). Therefore, we undertook a sensitivity analysis, where separate models were fitted with the minimum number of samples per herd per event varying from ≥2 to ≥10. In each model, covariates were included [herd-type, herd-size, numbers of animals bought into the herd during the year of testing, region (DVO)], and a herd-level random effect was included to control for repeated measures within herds. The adjusted odds ratio (aOR; 95%ci), the number of observations, and the number of herds represented in the dataset for each model was reported. We also assessed models in terms of discrimination via the AUC and calibration via the Hosmer-Lemeshow test (for fixed effects only).

Despite guidelines that recommend that MAP ELISA testing should occur at least 3 months following an animal's last TB skin test (AHWNI 2018), there was a potential for some samples to have been taken within this “risk window”. To investigate this potential effect at the herd event-level, we used available animal-level data on the timing of animal's last bTB test prior to the current sampling period (the week of the MAP/bTB test, as generally MAP samples were taken on the first day of a SCITT test). In total, there were approximately 54,000 animals and 85,000 MAP tests that we could associate with a bTB skin test prior to the current skin test/MAP event. Recent research from Ireland and the UK, suggests this risk window may be < 71 days (28, 39). Any MAP test events where one or more animals had a tuberculin test within a 71 day risk window were then excluded, and the final multivariable random effect model was refit and parameter estimates compared as part of the sensitivity analysis.

## Chronic Status

Milne et al. (31) and Milne et al. (32) used the episode file for bTB breakdowns to assign a status to herds based on herds experiencing problematic prolonged or recurrent breakdowns. Chronic cases were herds which experienced a prolonged (>365 days) or recurrent (1 breakdown of duration <1year, followed by two or more bTB breakdowns within 2 years) over a time-series 2004–2015 (32). We used this classification to stratify herds recruited to the present study. We also included a classification “Non-chronic episodic herd” for herds that experienced at least one episode during the time series that were non-chronic in nature. We investigated whether there was any relationship between MAP risk and chronic herd status.

## Results

### Summary Statistics

There were 10,219 observations (events) where one or more MAP tests occurred constituting 553 (5.4%) TB cases and 9,666 (94.6%) controls. MAP positive results were disclosed at 3,730/10,219 (36.5%) events. Cross-tabulations at the event level between TB cases/controls and predictor variables are presented in Table 1.

Overall, 43.0% of herds (1,933/4,500) exhibited at least 1 MAP serum positive sample over the study period. 9.2% of herds (414/4,500) experienced a bTB breakdown episode during a MAP testing event. Aggregating across the whole period of the study, 138 (3.1%) herds were deemed ‘chronic' breakdown herds (i.e., experienced at least one chronic breakdown over the period), 2,237 (49.7%) were non-chronic breakdown herds (i.e., experienced at least one breakdown over the period, but this breakdown did not constitute a chronic breakdown), and 2,125 (47.2%) did not experience an episode over the period of the study (i.e., clear of infection over the whole study period).

### Univariable Modeling

The unadjusted univariable associations between explanatory variables and the outcome is presented in Table 1. There was evidence of increased risk of a herd experiencing a bTB episode when disclosing with a MAP serum ELISA positive result (OR: 1.467 95%ci: 1.234–1.743), having an overall positive MAP status (serum plus additional feces data; OR: 1.476 95%ci: 1.243–1.754), being a dairy herd relative to a beef herd (OR: 1.957; 95%ci: 1.623–2.361), being in Armagh DVO relative to most other DVOs (referent in Table 1) with the exception of Newry (OR:1.116; 95%ci: 0.775–1.607) or Newtownards (OR: 1.356; 95%ci: 0.942–1.953). Increasing herd size was a risk factor (modeled as a linear predictor scaled to per 100 animals: OR: 1.308; 95%ci: 1.263–1.354), as was buying-in of cattle during the test year (linear predictor per 100 animals entering the farm: OR: 1.145; 95%ci: 1.087–1.207). There was little evidence of inter-annual variation in risk (overall categorical: *p* = 0.651), nor trends across years (modeled as linear: OR: 0.974; 95%ci: 0.940–1.009; quadratic: OR 0.998; 95%ci: 0.996–1.000; log(year): OR 0.891; 95%ci: 0.732–1.083).

### Multivariable Model

The final multivariable model (Table 2) did not include any interaction terms as there was no evidence that these terms improved the fit of the model (i.e., aORs straddled 0, AIC increased with term addition). The model exhibited an adequate discriminatory ability with an estimated AUC of 70.29 (95%ci: 68.10–72.47). Controlling for confounders, there remained evidence that the MAP status of a herd as assessed using serological testing was positively associated with bTB episode risk at the time of testing (aOR: 1.339; 95%CI:1.085–1.652; Table 2; including ancillary feces sample data: aOR: 1.356; 95%CI:1.099–1.673).

**Table 2:** Multivariable random effect binary logit model of the association between bovine TB (bTB) breakdown episode risk (outcome) and the *M. avium paratuberculosis* status assessed using ELISA serum testing (primary explanatory variable), while controlling for known bTB risk factors.

**Explanatory variable**	**aOR**	**SE**	**Lower 95%CI**	**Upper95%CI**
Serum neg.	ref.			
Serum pos.	1.339	0.144	1.085	1.652
Beef	ref.			
Dairy	1.141	0.183	0.834	1.562
Other/missing	0.877	0.202	0.558	1.377
Herd size (per 100 animals)	1.434	0.058	1.325	1.553
Buy in (per 100 animals)	1.203	0.052	1.105	1.309
Armagh	ref.			
Ballymena	0.692	0.315	0.284	1.687
Coleraine	1.162	0.353	0.640	2.109
Dungannon	0.970	0.287	0.544	1.731
Enniskillen	1.143	0.341	0.636	2.052
Larne	0.664	0.346	0.239	1.841
Derry/Londonderry	0.856	0.352	0.383	1.914
Newry	1.422	0.410	0.808	2.503
Newtownards	1.620	0.469	0.918	2.858
Omagh	1.211	0.333	0.707	2.075
Constant	0.008	0.002	0.005	0.015
ICC (ρ)^*^	0.426	0.040	0.351	0.505

* *LR test of ρ = 0: χ^2^ (df: 1) = 143.05; Prob > = χ^2^ < 0.001*.

### Sensitivity Analysis

**Figure 1:**
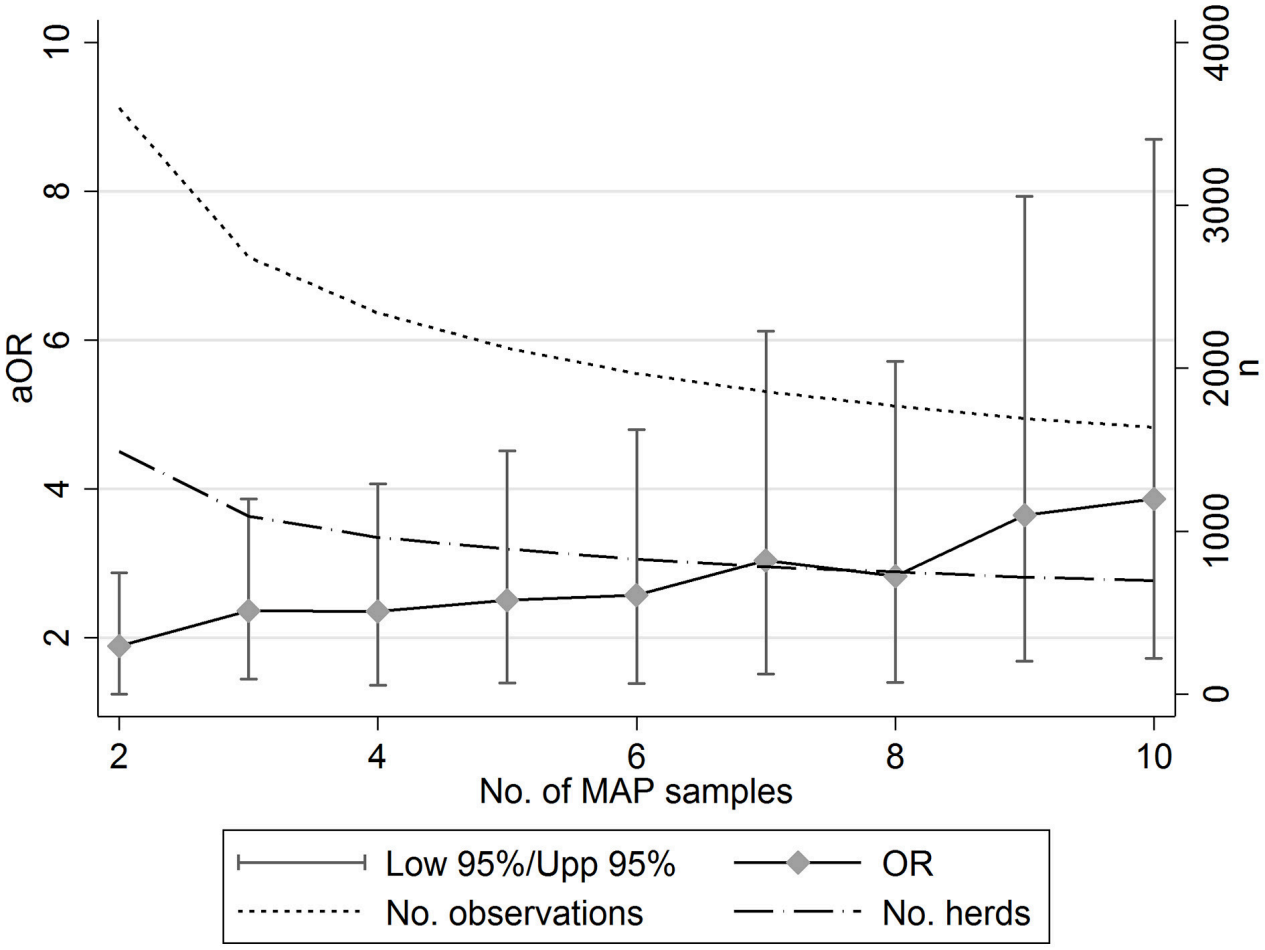
The relationship between the number of serum samples used (2+ to 10+ per sampling event) to assign a herd-level status for *Mycobacterium avium paratuberculosis* (MAP) and the estimated adjusted odds ratio (aOR) for increasing bovine TB risk (left hand y-axis). The corresponding number of observations and herds included in each model declined with increasing minimum samples taken (right hand y-axis).

The sensitivity analysis (Table 3; Figure 1) suggested that the effect size of this association increased as the number of samples used to inform the MAP status of herds increased. For example, a model fitted where a minimum of 2 samples was used to inform MAP herd status, suggested that bTB-MAP association had an aOR of 1.883. Increasing the minimum sample to 5 resulted in an aOR of 2.500, at ≥10 samples the aOR was 3.863. However, the overall sample (data set) size decreased with the increasing minimum number of samples required to assign a MAP status, and also the uncertainty in the parameter estimates increased (Figure 1). Despite this, the model's discriminatory ability generally improved with increasing minimum number of samples (minimum 1 sample AUC: 70.29; minimum of 10 samples AUC 75.80; Table 3), and calibration appeared not to be a large problem (based on HL on fixed effects; Hosmer-Lemeshow test: *p* > 0.2). It should be noted, that across all 10 models where minimum sample number was varied, the lower 95%CI aOR was always above 0, suggesting that the association was robust across the different subsamples of the population.

**Table 3:** Adjusted odds ratio (aOR) for a MAP positive herd experiencing a bTB herd breakdown episode, depending on the number of samples used per herd per event to categorize the herd's MAP status.

**Samples**	**Including risk window**	**Expl. Var**.	**aOR**	**SE**	**Lower 95%**	**Upper 95%**	**N**	**Herds**	**AUC**	HL-test^*^
≥2	Yes	MAP Serology	1.883	0.404	1.236	2.868	3,596	1490	73.31	0.267
≥2	No	MAP Serology	1.597	0.385	0.995	2.561	2,908	1373	73.91	0.447
≥3	Yes	MAP Serology	2.359	0.593	1.442	3.860	2,683	1093	73.61	0.500
≥3	No	MAP Serology	2.148	0.647	1.190	3.877	1,896	899	74.45	0.265
≥4	Yes	MAP Serology	2.351	0.657	1.359	4.066	2,340	963	74.12	0.301
≥4	No	MAP Serology	2.170	0.769	1.083	4.347	1,586	771	74.55	0.841
≥5	Yes	MAP Serology	2.500	0.753	1.386	4.511	2,124	892	74.82	0.570
≥5	No	MAP Serology	2.493	1.006	1.130	5.499	1,391	693	76.76	0.758
≥6	Yes	MAP Serology	2.571	0.818	1.379	4.796	1,968	828	74.82	0.106
≥6	No	MAP Serology	2.804	1.244	1.175	6.689	1,259	640	77.68	0.762
≥7	Yes	MAP Serology	3.038	1.084	1.509	6.114	1,857	782	75.80	0.367
≥7	No	MAP Serology	4.036	2.107	1.451	11.226	1,160	591	78.83	0.881
≥8	Yes	MAP Serology	2.827	1.015	1.399	5.713	1,769	751	74.82	0.350
≥8	No	MAP Serology	3.549	1.869	1.264	9.964	1,084	560	77.88	0.942
≥9	Yes	MAP Serology	3.648	1.445	1.679	7.929	1,692	719	75.49	0.512
≥9	No	MAP Serology	4.053	2.261	1.358	12.095	1,016	527	77.04	0.854
≥10	Yes	MAP Serology	3.863	1.600	1.716	8.699	1,638	697	75.80	0.526
≥10	No	MAP Serology	4.516	2.644	1.434	14.229	975	511	77.63	0.948

*These aORs were derived from models controlling for herd-type, herd-size, numbers of animals bought into the herd during the year of testing, region (based on District Veterinary Officer areas), and also controlled for repeated measures within the same herd (herd random effect)*.

* *This test was undertaken with fixed effects only*.

92.7% of MAP test events (9,477/10,219) were undertaken outside of the risk window (i.e., not < 71 days prior to samples tested). The sensitivity analysis (Table 3) suggested that excluding observations during the risk period increased the uncertainty in the parameter estimates (e.g., 2+ samples, excluding risk window obs: aOR: 1.597; 95%ci: 0.995–2.561). The magnitude of point estimate for association between MAP and bTB episode risk increased with increasing MAP samples used to assign herd MAP-status. However, this too resulted in an overall reduction in sample size. The highest AUC for any of the models during the sensitivity analysis was 78.83 for a dataset excluding observations within the risk window and having a minimum of 7 samples to inform the herd MAP status (aOR: 4.036; 95%ci: 1.451–11.226; *n* = 1160).

### Chronic TB Herd—MAP Associations

Table 4 presents data on the cross-tabulation of the herd's bTB classification (31, 32) and their MAP status based on serology ELISA testing. There was a marginally higher proportion of serum positive MAP observations for chronic herds (41.67%), than non-chronic episodic herds (39.85%), or bTB free herds (32.22%). The association at the univariable level was moderate and above 1 for the 95%ci (OR: 1.502; 95%ci: 1.185–1.906), however the evidence in support of this association was found to be weak when controlling for repeated measures (herd random effect; OR: 1.403; 95%ci: 0.717–2.747) and when controlling for confounders (RE plus confounders: aOR 1.170; 95%ci: 0.481–2.849). There were 34 herds which had MAP tests undertaken during chronic episodes. Removing these herds diminished the difference between the proportion MAP positive from chronic herds from 41.7% to 36.2%.

**Table 4:** Cross-tabulation of bTB herd classification and herd status for MAP using a serum ELISA test.

	**MAP serum**		
**Herd classification**	**Neg**.	**Pos**.	**Total**
Chronic episodic herd^*^	175	125	300
%	*58.33*	*41.67*	*100*
Non-chronic episodic herd^∧^	3,224	2,136	5,360
%	*60.15*	*39.85*	*100*
bTB free herds^$^	3,090	1,469	4,559
%	*67.78*	*32.22*	*100*
Total	6,489	3,730	10,219
%	*63.5*	*36.5*	*100*

* *A herd which experienced a prolonged (>365 days) or recurrent (1 breakdown of duration < 1 year, followed by two or more bTB breakdowns within 2 years) over a time-series 2004-2015 (32)*.

*^∧^A non-chronic herd that experienced at least one episode during the time series*.

$ *A herd which did not experience an episode during the time-series*.

## Discussion

This study identified a robust association between confirmed bovine TB breakdown episodic risk and herd MAP status, based primarily on MAP serum ELISA testing with some supplementary fecal testing. We found weaker evidence that there was an association between the long-term categorization of herds in terms of bTB risk (so called “chronic herds”). These data form part of an emerging evidence base to suggest that Johne's disease, caused by MAP infection, could be impacting on the dynamics of bTB in cattle herds, and consequently has a negative impact on bTB disease eradication (20–24).

Previous research from Spain has suggested that co-infection of MAP and bTB can lead to problems in clearing infection (23). In a bullfighting herd where there was a mixed infection, Alvarez et al. (23) showed that co-infection affected the sensitivity of the skin test. Indeed, other researchers have found during experimental co-infections that the performance of both the skin test (based on Purified Protein Derivative (PPD) tuberculin injection) and the interferon gamma test [e.g., (20, 30)] can be affected. However, the direction of effects are not always consistent across reported studies [e.g., (27, 40, 41)], but it appears that overall the current consensus is that co-infection reduces the sensitivity of tests for bTB (21), but may also negatively impact on the specificity (indeed, there is still some debate as to the dominant effect). Decreasing sensitivity of the CTT could result in more bTB false-negative animals disclosed in exposed herds, lowering the probability of truly infected herds being disclosed. Also for herds that do breakdown, a lowering of sensitivity increases the likelihood of infected animals being missed during whole herd tests, resulting in either lengthening of breakdowns or increasing the probability of recrudescence (31). If MAP decreases the specificity of the CTT, then more false positives would be disclosed. This could increase bTB disclosure rates (i.e., could result in uninfected herds being classed as experiencing a breakdown), however it should not be a major factor when post-mortem/laboratory confirmation of *M. bovis* infection is used to assign herd breakdown status (as in the case during the present study). Specificity interference could also affect the duration of breakdowns, as herds in the UK only get released from bTB breakdowns when the herd achieves clear CTT tests. In terms of epidemiological impact, specificity interactions have financial and resource attribution costs for control programs (i.e., the cost of culling uninfected herds, and keeping trading restrictions in truly uninfected herds), while sensitivity interactions have more direct consequences for disease eradication programs as truly infected animals may not be disclosed. It should be noted though, for bTB, multiple studies have demonstrated that the CTT achieves very high specificity [e.g., 100% (95% Bayes'CrI 99%, 100%) (9)], but moderate and variable sensitivity performance [e.g., 50% (95% Bayes' CrI: 26%, 78%; (9)].

TB in a herd can also affect the performance of the MAP ELISA serum tests [e.g., (27)], potentially increasing the number of false positives. Therefore, in our study, there was the risk that herd's MAP status could be affected by bTB presence. At the animal level, leaving a minimum period between skin testing with avian tuberculin can reduce interference of the ELISA blood test (AWHNI), but if the tested animal has been exposed to *M. bovis*, there is the potential for cross reactivity with the MAP ELISA test. We investigated the potential impact of MAP testing within a “risk window” prior to MAP testing during the sensitivity analysis. This work supported the idea that there may have been some impact of prior tuberculin testing on the association between TB episode risk and MAP status—pushing results toward the null. However, in models with greater MAP sampling (≥3 samples) and excluding observations within the risk window, there remained a strong positive association.

The effect of prior *M. bovis* exposure within herds (separate from just the tuberculin use alone) could still impact on the MAP test. While we believe this to be a risk for establishing causation in our study design, there are lines of evidence that suggest this risk to be low. There is 1. an annual testing regime of all animals in Northern Ireland for bTB, where all skin test reactors are culled (29), 2. follow-up stringent use of severe interpretation and interferon gamma testing is undertaken (6), 3. in some instances additional serological tests are deployed (42), 4. animals are managed so to reduce susceptible exposure to at-risk groups, and 5. is coupled with post-mortem surveillance. With this system, it is rare that disseminated infection is found in reactor animals [~45% have lesions, and most have very few lesions; (43)], and therefore transmission within herds is low, leading to small breakdown size [median <5 animals; (44)]. Therefore, the risk of MAP tested animals being exposed to *M. bovis* is low, even within breakdown herds. We utilized additional fecal data (culture and PCR) in an attempt to make our definition of MAP infection more robust, however we had limited fecal testing data available to inform our study (e.g., only 61 positive test result herds in our study).

As the impact of co-infection can impact on both diagnostics of bTB and MAP, and as MAP tests have low diagnostic sensitivity, we performed a sensitivity analysis on the number of samples used to infer a MAP status for herds. Interestingly, with increasing the numbers of samples (presumably increasing the sensitivity of the test (21), but also potential reducing the specificity of the MAP test) we found the association between the two pathogens increased, despite lowering the power to detect an effect (by reducing the overall *n*). As the serum ELISA MAP test has been shown to have reasonably high specificity for a screening test across studies [0.95–1.00; (37)], and as we sampled typically small numbers of animals per herd (mean 10), we do not anticipate that MAP false positive results would be the major factor influencing our inferences.

This work does add some evidence that exposure to non-tuberculosis bacteria could be having an effect in Northern Ireland (26, 30, 43). Barry et al. (30) showed using experimental animal models that co-infection could affect the gamma interferon test (but not necessarily the skin test). Using field data, our group previously found that animals with large avian tuberculin reactions (but CTT negative) had an increased risk of being TB confirmed and having greater number of lesions at slaughter, relative to non-reactor animals (26, 43). Such animals were potentially exposed to non-tuberculosis mycobacteria (including environmental exposure or MAP). Therefore, there was a risk that some co-infected animals were missed during antemortem TB testing. Coupled with the present study, there is a strong argument for further investigation into the mechanism by which MAP-bTB-coinfection is impacting on the bTB statutory eradication program.

Our models used a parsimonious base model from which we tested our co-infection hypothesis. This base model confirmed that herd size, the act of buying in of cattle, regional variation (likely representing spatial variation in underlying infection pressure) are all very important explanatory factors for bovine TB risk (34, 45). However, despite our previous work [e.g., (6, 31, 46)], there was less evidence that major enterprise type was very important in our final model (though invariably associated with the outcome). This could be related to our ability to consistently categorize herd types in Northern Ireland. In the present study we used two pieces of data available to the study, herd type and having a milk license. Both are recorded within the APHIS dataset, and both are known to have limitations. For example, where farms change their enterprise type, the recording of this change can be delayed within the dataset. Furthermore, where there are mixed enterprises, it is difficult to assign a single category. Despite this, we have previously reported differential risk at both the herd [e.g., (44)] and animal level [e.g., (43)] in Northern Ireland using similar datasets. This covariate was not our primary interest during this study, however further more refined “real-time” classification of herds is required to improve epidemiological modeling in Northern Ireland.

## Limitations

Our models were based on retrospective observational datasets that were not collected for this specific purpose, and therefore the population they represent may not be fully representative of herds in the broader population in Northern Ireland. Indeed, inclusion criteria for the study was for voluntary participation in a MAP control/surveillance scheme in Northern Ireland, which may have introduced some selection bias. Furthermore, the median number of samples taken per event was 1, and therefore these animals are more likely representative of ‘suspect' cases, sampled as part of a veterinary investigations (though this is not the case for CHS herds). Indeed, therefore, there may have been some directed sampling toward the highest risk animals within the cohort. A further problem with the low number of animals tested per event is that, because of the sensitivity of the MAP test, there was the risk of misclassifying the herds. However, we addressed this with our sensitivity analysis. There was the overall problem of assessing co-infections at the herd level, though they have been undertaken previously [e.g., (47)]. Obviously, herds are a dynamic collection of animals, which can move in (buying in, birth) or out (market, abattoir, death) of herds. Therefore, finding associations at this level of organization is probably more biased toward the null than animal-level experimental analyses where there is little ambiguity as to exposure to either or both pathogens. Despite the limitations of this study, we believe there are useful associations to build further inferences about the dynamics of two difficult chronic pathogens of cattle herds.

## Conclusion

We have found robust associations between bTB herd breakdown episode risk and concurrent MAP infections at the herd level. This association was positive, indicating that breakdown herds exhibited a higher risk of co-infection than sampled herds which were not experiencing a breakdown. Furthermore, there was a marginally higher proportion of chronically infected herds with evidence of MAP infection than bTB free herds over the study period. During the study we investigated whether the sampling frequency of herds to determine MAP status, and the time-period between a previous tuberculin test and MAP test, had an impact on these associations. Increasing sampling frequency increased the magnitude of the point estimate associations and the uncertainty around these estimates, while decreasing *N*. The sampling of animals within a “risk window” where cross-reactivity may occur after a TB tuberculin test did appear to impact the association by increasing uncertainty and lowering the magnitude of the association. The exact mechanism for our findings cannot be readily determined with these data, however we hypothesize given results from elsewhere that MAP may be masking bTB infection in herds experiencing TB breakdowns. This masking effect may impact on the ability to identify exposed cattle, cull them, and thus clear infection. Further research is required to better understand how these patterns emerge, and whether (or which) co-management tools would yield optimal control benefits.

## Author Contributions

AB conceived and designed the study, project managed, undertook data analysis, and interpretation of data for the paper. AB drafted the initial manuscript and revised it critically. JG managed all research databases contributing to the work, and was involved in data merging, validation, and cleaning, and contributed to data interpretation. GM was involved in generating the chronic herds data set and episode file, contributed to the data interpretation, and critically revised the manuscript. MG-G led and coordinated the AFBI cattle health scheme (2010–2018) which contributed data, managed the diagnostic laboratories undertaking MAP testing, contributed to data interpretation and study design, and critically revised the manuscript. SS was involved in the initial concept for the work, managed a Johne's control program (AHWNI) in Northern Ireland which contributed data, contributed to data interpretation and study design, and critically revised the manuscript. All authors read and made comments and/or edits to the final manuscript.

### Conflict of Interest Statement

The authors declare that the research was conducted in the absence of any commercial or financial relationships that could be construed as a potential conflict of interest.
