# Supplementary material for: Is There a Relationship Between Bovine Tuberculosis (bTB) Herd Breakdown Risk and Mycobacterium avium subsp. paratuberculosis Status? An Investigation in bTB Chronically and Non-chronically Infected Herds
Source: Front Vet Sci. 2019 Feb 14;6:30. doi: 10.3389/fvets.2019.00030 (PMC6382694; doi:10.3389/fvets.2019.00030)
Supplement: Supplementary file 1 [file Data_Sheet_1.PDF]

Supplementary material

Supplementary Material 1: Additional Johne's Disease laboratory methods

Extraction

-Inactivated **culture** extracted using the APPLIED BIOSYSTEMS MagMAX™ AM1836 bead-based sample disruption protocol on partially automated magnetic nucleic acid particle processors. (MagMAX™ -96 viral isolation kit).

- **Faeces** DNA extracted as per the APPLIED BIOSYSTEMS MagMAX™ AM1840 bead-based sample disruption protocol on partially automated magnetic nucleic acid particle processors. (MagMAX™ total Nucleic Acid isolation kit)

PCR testing- testing carried out initially by Adiavet ParaTB real time PCR (supplied by Bio Merieux), and during 2016 onwards using the VetMAX kit (Taqman real time PCR). Real time PCR is run as a duplex assay. Each sample is simultaneously tested for both MAP DNA and 'Xeno™ DNA' control.

Controls

IC- Xeno is added at the extraction stage and is the internal control (the absence of xeno signal will invalidate a negative MAP result as it will be a sign of the presence of PCR inhibitors in the sample).

Negative controls – 1 water sample extracted for every 10 samples tested.

-No template control -1XNTC per assay.

Positive controls- 2X positive per assay. Positive control provided by manufacturer 'MAP control DNA (3000 copies/µl)'. Ct values are monitored and considered when determining acceptability.

Below are the criteria used when authorising a MAP assay.

Expected Ct values for MAP

Reaction	MAP Ct
MAP positive sample	<37
MAP negative sample	No signal
MAP positive control	28-34
Inconclusive result	≥ 37<40*
NTC	No signal
Water (negative control)	No signal

* Inconclusive results may result from low positive samples or cross contamination.

Expected Ct values for Xeno DNA

Reaction	IC RNA Ct
MAP positive sample	29-35**
MAP negative sample	29-35
MAP inconclusive sample	29-35
MAP positive control	27-34
No template control	No signal
Water (negative control)	29-35

** Samples containing high level of MAP DNA may result in high Xeno DNA Ct values.

Other QA measures employed included the use of a Laboratory Information Management System (assays upon completion are exported electronically to LIMS for subsequent authorisation and reporting). Internal quality assurance (IQA) data, such as batch numbers of kits and controls, is also recorded on LIMS for each run.

2) The only deviation from the manufacturer's recommendation is the reduction of mastermix volumes by one third (see table below).

	Volume per reaction (µl)	
Component	Original	One third volume (1:3)
2xqPCR Mastermix	12.5	4
25xMAP Primer Probe mix	1	0.3
Nuclease-free water	3.5	1
DNA template	8	3
**Total volume**	**25**	**8.3**
